# Immunobiology of a rationally-designed AAV2 capsid following intravitreal delivery in mice

**DOI:** 10.1038/s41434-023-00409-x

**Published:** 2023-06-29

**Authors:** Michael Whitehead, Andrew Sage, Tom Burgoyne, Andrew Osborne, Patrick Yu-Wai-Man, Keith R. Martin

**Affiliations:** 1 John Van Geest Centre for Brain Repair, Department of Clinical Neuroscience, University of Cambridge, Cambridge, UK; 2 Division of Cardiovascular Medicine, University of Cambridge, Addenbrooke’s Hospital, Hills Road, Cambridge, UK; 3 UCL Institute of Ophthalmology, London, UK; 4 Paediatric Respiratory Medicine, Primary Ciliary Dyskinesia Centre, Guy’s and St Thomas’ NHS Foundation Trust, London, UK; 5 MRC Mitochondrial Biology Unit, Department of Clinical Neurosciences, University of Cambridge, Cambridge, UK; 6 NIHR Biomedical Research Centre at Moorfields Eye Hospital and UCL Institute of Ophthalmology, London, UK; 7 Wellcome Trust-MRC Cambridge Stem Cell Institute, University of Cambridge, Cambridge, UK; 8 Centre for Eye Research Australia, Royal Victorian Eye and Ear Hospital, Melbourne, VIC Australia; 9 Ophthalmology, Department of Surgery, University of Melbourne, Melbourne, VIC Australia

**Keywords:** Gene therapy, Immunology

## Abstract

Adeno-associated virus serotype 2 (AAV2) is a viral vector that can be used to deliver therapeutic genes to diseased cells in the retina. One strategy for altering AAV2 vectors involves the mutation of phosphodegron residues, which are thought to be phosphorylated/ubiquitinated in the cytosol, facilitating degradation of the vector and the inhibition of transduction. As such, mutation of phosphodegron residues have been correlated with increased transduction of target cells, however, an assessment of the immunobiology of wild-type and phosphodegron mutant AAV2 vectors following intravitreal (IVT) delivery to immunocompetent animals is lacking in the current literature. In this study, we show that IVT of a triple phosphodegron mutant AAV2 capsid is associated with higher levels of humoral immune activation, infiltration of CD4 and CD8 T-cells into the retina, generation of splenic germinal centre reactions, activation of conventional dendritic cell subsets, and elevated retinal gliosis compared to wild-type AAV2 capsids. However, we did not detect significant changes in electroretinography arising after vector administration. We also demonstrate that the triple AAV2 mutant capsid is less susceptible to neutralisation by soluble heparan sulphate and anti-AAV2 neutralising antibodies, highlighting a possible utility for the vector in terms of circumventing pre-existing humoral immunity. In summary, the present study highlights novel aspects of rationally-designed vector immunobiology, which may be relevant to their application in preclinical and clinical settings.

## Introduction

Adeno-associated virus serotype 2 (AAV2) is a gene therapy vector that can be used to deliver therapeutic genes to diseased cells in the retina [1]. The platform is favoured for its relatively low immunogenicity and capacity to induce long-term therapeutic transduction in non-dividing neural tissue [2]. This was evidenced in 2017 by the regulatory approval of Luxturna (voretigene neparvovec-rzyl), an AAV2-based gene therapy that delivers a functional copy of the *RPE65* gene to retinal pigment epithelium (RPE) cells in the outer retina via subretinal injection (SRT) [3]. More recently in 2020, scientists and clinicians investigating GS010 (lenadogene nolparvovec) reported improvements in visual acuity after intravitreal injection (IVT) of AAV2.*ND4* to patients diagnosed with Leber hereditary optic neuropathy (LHON), a mitochondrial genetic eye disorder caused by the m.11778 G > A point mutation in the *MT-ND4* gene, which is characterised by rapid loss of retinal ganglion cell (RGC) function [4].

Despite these recent successes, researchers have sought to improve AAV2 vectors across multiple parameters, including their transduction capacity, ability to evade pre-existing neutralising antibodies (NAbs), genome packaging capacity, and functional viral titres during manufacturing at scale for in-human trials [5]. A comprehensive literature now exists detailing efforts to improve AAV2 transduction potential via capsid mutagenesis, that is, substituting wild-type residues for those with desirable properties [6]. One example of this involves the mutation of phosphodegron residues that are thought to be phosphorylated by cytosolic protein kinase enzymes, thus facilitating ubiquitin-dependent degradation of AAV2 virions. Switching these residues to amino acids that are refractory to phosphorylation has been shown to increase target cell transduction, possibly by augmenting nuclear transfer of vector genomes [7, 8]. The reduction in cytosolic degradation of AAV2 has also been studied in the context of CD8 T-cell responses to infected cells. The authors demonstrated that a reduction in the presentation of AAV2 capsid antigen on major histocompatibility complex class I (MHC c. I) correlated with attenuated cytolytic killing of infected hepatocytes by adoptively transferred CD8 T-cells following systemic vector administration in a *rag* KO mouse (which exhibited abrogated endogenous B- and T-cell function) [9]. Boye et al. also showed that triple, quadruple and quintuple phosphodegron mutant capsids demonstrate attenuated binding affinity to AAV2’s primary attachment receptor, heparan sulphate proteoglycan (HSPG), which may explain their capacity to permeate deeper into retinal tissue, possibly due to reduced sequestration in the inner limiting membrane (ILM) that demarcates the vitreo-retinal interface [1]. These findings therefore demonstrated that the in vivo properties of phosphodegron mutant AAV2 may be explained by factors in addition to reducing cytosolic degradation.

Some literature reports have described the eye as partially immune-privileged due to physical barriers like the blood-retinal-barrier (BRB) and an apparent lack of efferent lymphatics, and expression of anti-inflammatory factors [10]. However, AAV2 delivery via IVT has been associated with the infiltration of mononuclear cell infiltrates into the eye [11], in addition to a possible role for dendritic cell (DC) and macrophage infiltration, and the proliferation of microglia cells [12]. Another publication observed AAV8 genomes in blood, splenic and lymphatic samples post-IVT, and suggested a possible role for vector shedding in facilitating systemic immune responses to vector protein via the IVT route of administration [13]. Collectively, these findings question the role of ocular immune privilege in AAV-mediated gene therapy studies and suggest investigations to better understand the nature and clinical relevance of the immune response to wild-type and mutant AAV capsids are warranted.

Here, we demonstrate that a triple phosphodegron mutant AAV2 capsid is associated with increased humoral immune activation, retinal T-cell infiltration, splenic germinal centre reactions and DC activation, and retinal gliosis when compared to wild-type AAV2 particles. However, we did not detect electrophysiological perturbations after vector administration. We also show that the triple mutant capsid is more resistant to neutralisation by soluble heparan sulphate (HS) and anti-AAV2 NAbs compared with wild-type AAV2, highlighting a possible utility for phosphodegron mutant AAV2 vectors in circumventing pre-existing NAb responses. In summary, the present study highlights novel aspects of phosphodegron mutant AAV2 immunobiology relevant to both preclinical and clinical application.

## Methods

### Vector production

Mutagenesis of *rep/cap* gene plasmids was performed at Vector Biolabs (Vector Biolabs 293 Great Valley Parkway, Malvern, PA 19355, USA). The correct substitutions were confirmed by Sanger Sequencing. AAV2 vectors were manufactured at ViGene Biosciences (9430 Key West Avenue, Suite 105, Rockville, MD 20850, USA). For AAV2 manufacturing, a standard triple plasmid transfection protocol was used (pAAV-CAG-GFP, pHelper, pRep2/Cap2). Vectors were extracted from HEK-293T cells via repeated freeze-thawing, purified using iodixanol gradient ultracentrifugation and suspended in phosphate-buffered saline (PBS). All vector preps were titred by ViGene via qPCR with primers specific to the AAV ITR regions and the SYBR Green detection method. Viral preps were treated with a DNAse I followed by digestion with Proteinase K prior to qPCR analysis. All vectors were aliquoted upon arrival and stored at –80 °C until further use.

### Use of animals

All procedures performed on animals were approved by the UK Home Office in accordance with the UK Animals (Scientific Procedures) Act, and undertaken in accordance with the Association for Research in Vision and Ophthalmology’s (ARVO) Statement for the Use of Animals in Ophthalmic and Visual Research. The sample size used in each experiment is stipulated in the figure legends.

### In vivo study design

We performed bilateral intravitreal injections of 2E8 genome copies (GC)/eye AAV2, 2E8 GC/eye phosphodegron mutant AAV2, or PBS vehicle into C57BL6/J adult male mice. After a three week incubation period, electroretinography was performed. Blood samples were also taken by cardiac puncture for serological testing, before eyes were enucleated and fixed for immunohistochemical analysis. The spleens of mice were also extracted for analysis via flow cytometry. A summary of the in vivo study design is given in Fig S1 (Supplementary Materials).

### Intravitreal (IVT) vector injection

For anaesthesia, 50 mg/kg ketamine and 10 mg/kg xylazine were delivered through intraperitoneal (IP) injection. 1% tetracaine solution (Bausch & Lomb) was also used as a local anaesthetic and pupils were dilated with 1% tropicamide to visualise correct placement of the needle in the vitreous. Vectors were diluted in sterile PBS to obtain the desired vector concentration in 2 μL solution. For bilateral IVTs, a 5μL Hamilton Syringe (#65RN; Needle: 33 G, 8 mm, point style 2, Hamilton Co) was used to puncture the sclera approximately 1 mm posterior to the superior-temporal limbus and inject the vector solution into the vitreous. Solution was injected slowly over a 30 s period to prevent sudden increases in intraocular pressure. The cornea was then punctured with a 30 G needle to limit reflux of the injection solution from the site of injection. Animals were recovered in a warm cabinet and monitored every 15 min until fully recovered. To collect tissues, mice were sacrificed using the Schedule 1 method of cervical dislocation. Eyes would be excluded from analysis if (i) the injection score was graded ‘poor’ e.g. due to significant reflux of injected solution, or (ii) a cataract developed.

### NAb assays

HEK-293T cells were seeded at 10,000 cells per well in white-walled, clear-bottomed 96-well plates (Sigma Aldrich) and used in the neutralising antibody (NAb) assay when 80% confluent. Blood sera samples were collected via saphenous vein bleeds or cardiac puncture, allowed to clot overnight at 4 °C, then centrifuged at 300 RCF. The resulting supernatant was designated sera and stored at –20 °C until further use. Two- or three-fold serial dilutions of serum samples were prepared in DMEM in a 96-well plate. AAV2.CMV.Luciferase was added to a final concentration of 1E9 GC/mL, and incubated for 1 h to allow the anti-AAV NAbs to bind to the vectors. Sera-AAV mixtures were then transferred to the HEK-293T cultures and incubated for 24 h. Luminescent signal was detected by aspirating the media and adding 25 µL of BrightGlo assay substrate (ProMega), covering the plates with foil and incubating for 5 min. Luminescence was recorded using a FLUOstar Omega plate reader, and IC50 (dilution of sera that yielded a 50% reduction in the luminescent signal) and area-under-the-curve (AUC) was also calculated in GraphPad Prism.

### Total antigen binding antibody assays

To assess total binding antibody (TAb) levels, an enzyme-linked immunosorbent assay (ELISA) was used. 96-well plates were coated with 50 µL 1E10 GC/mL AAV2.CAG.GFP for 2 h at room temperature. AAV was aspirated and plates were blocked with a 5% milk powder 0.2% tween-20 solution for 2 h at room temperature. Serum samples were diluted 1:2,000 in blocking solution and added to the wells in a 100 µL volume. Plates were incubated overnight at 4 °C, followed by one wash in blocking solution and two washes with tris-buffered saline (TBS). Horseradish peroxidase (HRP)-conjugated secondary antibodies were diluted to a final concentration of 1:50,000 in TBS, added to the wells and incubated for 2 h at room temperature. Plates were washed three times with TBS, followed by addition of 50 µL 3,3’,5,5’-tetramethylbenzidine (TMB) substrate, and a 30 min incubation at room temperature. Signal detection was performed on a FLUOstar Omega plate reader, measuring absorbance at 650 nm.

### Retinal flatmounts

Eyes were enucleated and immersed in ice-cold 4% paraformaldehyde (PFA) for 24 h. Under a dissecting microscope, the cornea and lens were removed and the retina was dissociated from the eye cup using forceps. Four incisions were then made from the peripheral retina to within 0.5 mm of the optic nerve head to allow the retina to flatten. Retinas were washed three times in PBS, then blocked with 3% normal goat serum (NGS), 1% bovine serum albumin (BSA), and 0.3% Triton X-100 diluted in PBS for 1 h at room temperature. Primary antibodies were diluted to the concentrations stated below (Table 1) in blocking solution and incubated with retinas overnight at 4 °C. Samples were washed three times in PBS. Alexaflour-conjugated secondary antibodies and 2-(4-amidinophenyl)-1H-indole-6-carboxamidine (DAPI) were diluted in PBS 1:500 and 1:5,000 respectively and incubated with retinas for 2 h at room temperature. Imaging and analysis of GFP was performed without antibody-mediated signal amplification. Retinas were washed three times in PBS and mounted onto Superfrost Plus slides (VWR) with RGCs facing upwards, using FluoSave (Merck Millipore) to preserve the fluorescent signal. To obtain tilescan images of the whole flatmounted retina, a Leica DMi8 microscope was used and set to a 20x objective. An SPE confocal microscope equipped with a 40x magnification (Leica Microsystems) was used to obtain high magnification representative images. For quantification of RBPMS and GFP co-localisation, eight images per retina were taken using a 20x objective and assessed using Volocity Software. Assessment of astrocyte dendritic arbour complexity was performed in ImageJ using the Skeletonise/Analyse Skeleton functions.

**Table 1:** List of primary antibodies used in immunohistochemical analysis of flatmounted retina tissue.

Target	Species	Dilution	Vendor	Catalogue number
RBPMS	Guinea pig	1:500	PhosphoSolutions	1832-RBPMS
GFAP	Rabbit	1:500	Dako	Z0334
Tuj1	Mouse	1:500	Biolegend	MMS-435P
Iba1	Guinea pig	1:250	Synaptic Systems	234 004

### Retinal cryosections

Following enucleation, eyes were immersed in 4% PFA for 24 h at 4 °C, dehydrated in 30% sucrose for 24 h at 4 °C, and embedded in optimal cutting temperature compound (OCT; Sakura Finetek). 13μm tissue sections (through the dorsal-ventral/superior-inferior axis of the retina) were prepared using a Bright OTF 5000 cryostat (Bright Instruments) and Superfrost Plus slides (VWR). For some staining protocols (see Table 2 below), citrate buffer-mediated antigen retrieval was used. Slides were incubated in 90 °C sodium citrate buffer (Abcam) for 30 mins, allowed to cool to room temperature, then washed twice in PBS. Tissue sections were blocked and permeabilised with 10% NGS, 0.5% Triton X-100 and 0.5% BSA diluted in PBS for 1 h at room temperature. Primary antibodies were diluted in 5% NGS, 0.5% Triton X-100 and 0.5% BSA to the concentrations stated below and incubated overnight at 4 °C. Table 2 outlines all the primary antibodies used for immunohistochemical analysis of retinal cryosections. Samples were washed three times with PBS for 10 mins, then incubated with secondary antibodies and DAPI diluted 1:1,000 and 1:5,000 respectively for 2 h at room temperature. Samples were washed three times in PBS and a glass coverslip was mounted with FluoSave reagent (Merck Millipore). For imaging, a Leica DM6000 epifluorescence microscope was used to obtain 8–12 images per sample for quantification of fluorescence intensity. Quantification of fluorescence intensity per image was performed in ImageJ. Images were thresholded and measurements of integrated density were taken per field-of-view (FOV) and for each region-of-interest (ROI). For glial fibrillary acidic protein (GFAP) analysis, Single Neurite Tracer, an ImageJ plugin, was used. Each observable GFAP+ fibril per FOV was traced manually on the software, which then calculated the integrated density of fluorescence and length of each fibril. Counting of CD4 and CD8 T-cells was performed using the Analyse Particles function.

**Table 2:** List of primary antibodies used in immunohistochemical analysis of cryosectioned retina tissue.

Target	Species	Dilution	Vendor	Catalogue number	Antigen retrieval
RBPMS	Rabbit	1:500	Abcam	ab152101	No
GFAP	Rabbit	1:500	Dako	Z0334	No
Iba1	Guinea pig	1:250	Synaptic systems	234 004	No
CD4	Rabbit	1:250	Abcam	ab183685	Yes
CD8	Rabbit	1:1000	Abcam	ab217344	Yes

### Flow cytometry

Spleens were extracted and processed into a single cell suspension by passing through a 70 µm cell sieve using a syringe plunger. Red blood cells (RBCs) were first lysed with ammonium chloride RBC lysis buffer, and then 1-2E6 cells were stained with a cocktail of fluorescently-tagged antibodies and a live-dead stain (Zombie aqua, Biolegend). After washing, cells were fixed with formaldehyde solution (BD Cellfix) and filtered through a 30 µm filter before analysis on a LSRII Fortessa flow cytometer (BD). Prior to antibody gating, live single cells are gated based on FSC, SSC and negative live-dead staining. A list of the antibodies used for these analyses is given below (Table 3).

**Table 3:** List of antibodies used for flow cytometry analysis.

Target	Fluorophore	Clone	Vendor
CD19	BV650	6D5	Biolegend
IgM	APC	II/41	ThermoFisher
IgD	PerCPVio700		Miltenyi
CD95	PE-Cy7	Jo2	BD Biosciences
GL7	eF450	Ly-77	Biolegend
MHC c. II	APCVio770		Miltenyi
CD3	AF488	145-2C11	Biolegend
CD4	AF700	RM4-5	Biolegend
CD8	PerCP	53-6.7	Biolegend
CXCR5	PE	L138D7	Biolegend
PD-1	PE-Cy7	4B12	Biolegend
CD44	BV605	1M7	Biolegend
CD62L	PacBlue	MEL-14	Biolegend
CD25	APC	PC61	Biolegend
CD11c	PE-Cy7	N418	Biolegend
CD8a	PerCP	53-6.7	Biolegend
CD11b	AF488	M1/70	Biolegend
XCR1	APC	MPC-11	Biolegend
SiglecH	PE		Miltenyi

### In silico modelling of selected mutations

To visualise VP3 AAV2 capsid monomer and oligomers in silico, 6ih9, a 2.8 Å resolution cryoelectron microscopy-derived structure, was downloaded from the Protein Data Bank. Both monomeric and oligomeric forms of 6ih9 were visualised using PyMol software. To depict the mutated capsid residues, PyMol’s internal mutagenesis wizard was used. The residue and the appropriate substitute residue was selected, then the correct rotameric conformation of the mutated amino acid was chosen in accordance with PyMol’s prediction. Mutated residues were coloured Y444F, red; K556E, green; S662V, yellow. Heparan binding domains (HBDs; R484, R487, K532, R585, and R588) were coloured in blue. AAV receptor binding domains (AAVR BDs; R471, D528, Q589, T592, S262, Q263, G265, A266, S267, N268, H271, N382 and Q385) were highlighted in orange. A Radial Interpretation of Viral Electron Density Map (RIVEM) was also produced. This programme reads atomic coordinates from Protein Data Bank files, and converts these into a stereographic projection.

### Assessment of in vitro transduction efficiency and neutralisation by HS and anti-AAV2 NAbs

HEK-293T and ARPE-19 cells were cultured on poly-L-lysine (10 µg/mL; Sigma Aldrich) coated plates. Cells were incubated in Dulbecco’s Modified Eagles Medium (DMEM) supplemented with 10% foetal bovine serum (FBS) and 1% penicillin/streptomycin (P/S) until 80% confluent. For transduction with vectors, cells were washed with PBS, then incubated with vectors diluted to stated concentrations in serum-free DMEM. Cells were passaged in T75 flasks in a 37 °C 5% CO_2_ incubator. Media was replaced every two days and cells were split 1:10-1:20 when they had reached 80–90% confluency. Cells were counted using haemocytometry with trypan blue live dead staining, and cultures were only used for experiments if viability exceeded 90%. To determine the efficiency of cellular transduction, cells were detached with TrypLE (Thermo Fisher) for 5 min in the incubator. TrypLE was deactivated by addition of DMEM + 10% FBS + 1% P/S, and cells were centrifuged at 300 RCF for 5 min. The supernatant was aspirated and the remaining pellet was fixed in 150 µL 4% PFA. To assess the number of transduced GFP+ cells, an Acurri C6 flow cytometer was used. Forward (FSC) and side scatter (SSC) gating was used to identify an appropriate population for analysis. An FSC/FITC-A gate was used to identify GFP+ cells, and the population with a FITC-A fluorescence intensity of >10^4 RFUs was deemed to be expressing GFP.

To determine the neutralisation of wild-type and mutant AAV2 capsids in the presence of HS, 4000 AAV2 GC/ng HS was mixed and incubated at room temperature for 1 h before addition to HEK293T cell cultures. To study the neutralisation of wild-type and mutant AAV2 capsids by anti-AAV2 NAbs, wild-type and mutant AAV2 were incubated with anti-AAV2 NAb containing sera for 1 h before addition to HEK293T cell cultures. To assess differences between groups, the remining infectivity (I/I0) was calculated by normalising the values for neutralised wild-type and mutant AAV2 to no-sera or no-HS control groups.

### Electroretinography

Electroretinography (ERG) was performed using a Diagnosys ColorDome LabCradle machine. Mice were dark adapted in a closed cabinet for 18–24 h before the ERG procedure. For anaesthesia, 50 mg/kg ketamine and 10 mg/kg xylazine were delivered via IP. An eyedrop of 1% tetracaine solution was used as a local anaesthetic and pupils were dilated with 1% tropicamide and 2.5% phenylephrine hydrochloride. Before placement of electrodes, eyes were checked for cataracts or absence of a red reflex, which would result in exclusion of that particular eye. A grounding electrode was placed into the tail, and the reference electrodes were placed around the eyes. The recording electrodes were then placed very gently onto the apex of the cornea. A small drop of lacrilube was then applied to the cornea using a 1 mL syringe/30 g needle to couple to the recording electrodes to the eye. The protocol used had 17 steps, including 7 positive scotopic threshold responses (pSTRs; measure of retinal ganglion cell function), 4 B-waves (rod bipolar cell function) and 4 A-waves (rod photoreceptor function).

### Statistical analysis

Normality of distribution was assessed with a Shapiro Wilk test. If the data did not meet this assumption, non-parametric Kruskal-Wallis and Dunn’s posthoc tests to assess more than two groups, or a Mann–Whitney test to compare two groups. The homogeneity of variance was also assessed, and for data that was normally distributed but exhibited heteroskedasticity, a Brown-Forsythe ANOVA and Dunnett’s T3 posthoc test was used to compare more than two groups, and a Welch’s t-test to compare two groups. If datasets were normally distributed and exhibited homogeneity of variance, ordinary one-way ANOVA and Tukey or Dunnett’s posthoc tests (more than two groups) or a Student *t* test (two groups; two-tailed, unpaired) was used.

## Results

### Transduction profiles of selected phosphodegron mutations in vitro and in vivo

**Fig. 1:**
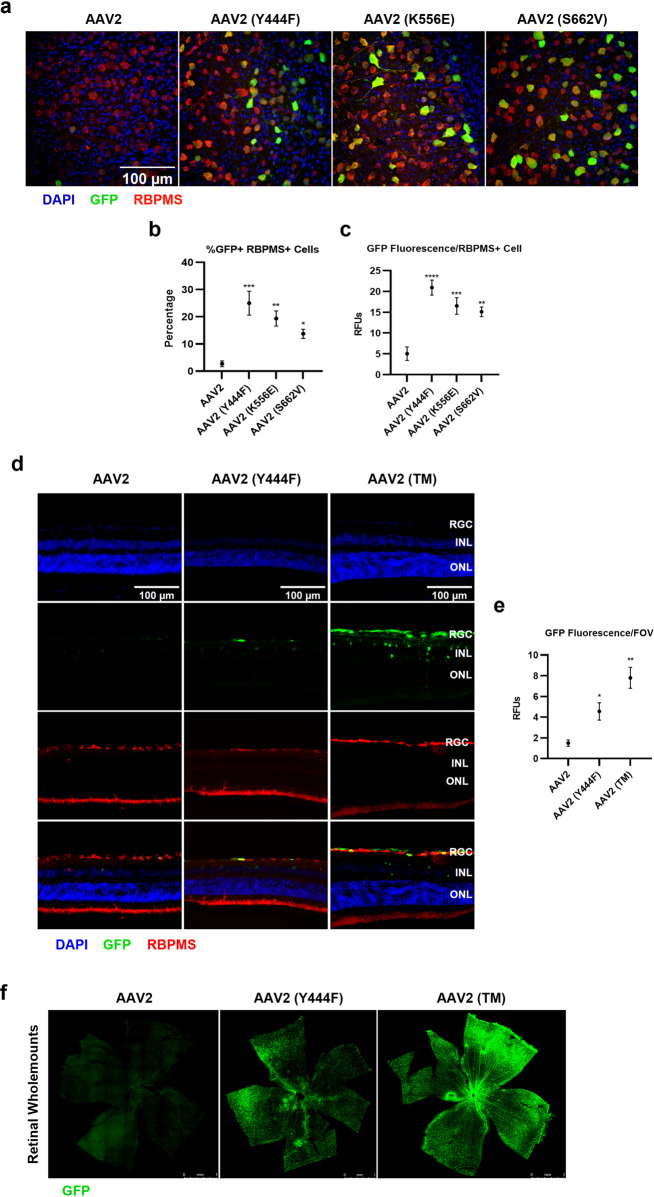
A combination of three phosphodegron mutations leads to a synergistic increase in transduction of the murine retina. Vectors were injected intravitreally and tissue was taken for analysis after three weeks. All data is presented as a column graphs showing the mean value for each group ±SEM. All statistical analyses are vs. the AAV2 group. **a** Representative images of retinal wholemounts depicting increased levels of GFP in the phosphodegron mutant groups compared to AAV2 WT control three weeks after bilateral intravitreal injection of 2E8 GC/eye. Quantification of GFP expression levels in wholemounted retina samples was performed in Volocity. **b** Percentage of GFP + RBPMS+ cells, and (**c**) mean GFP immunofluorescence/RBPMS + ROI/FOV was calculated. *p < 0.05, **p < 0.01, ***p < 0.001, parametric ANOVAs and Dunnett’s posthoc tests, *n* = 6–8. **d** Representative 40x epifluorescent microscope images of retinal sections transduced with AAV2, or phosphodegron mutant capsid AAV2. RGC retinal ganglion cell layer, INL inner nuclear layer, ONL outer nuclear layer. Quantification was performed in ImageJ. Here, (**e**) mean GFP fluorescence levels/FOV was calculated. *p < 0.05, **p < 0.01, ****p < 0.0001, Kruskal-Wallis and Dunn’s posthoc tests, *n* = 6–8. **f** Representative tilescan images showing wholemounted retinas from each group.

Three phosphodegron mutations, Y444F, K556E and S662V, were selected for analysis in accordance with previous reports [6, 7]. First, the transduction potential of each vector was assessed in vivo via IVT to C57BL6/J mice. Each vector was found to induce higher levels of GFP expression in RBPMS+ RGCs compared with wild-type AAV2 (Fig. 1a–c). Next, the three mutant residues were incorporated into a single capsid, hereafter referred to as AAV2 (TM). AAV2 (TM) induced higher levels of GFP expression in HEK-293T and APRE-19 cell cultures when assessed with flow cytometry in vitro (Supplementary Fig. 1a–c). These observations translated into an in vivo setting, where AAV2 (TM) IVTs were associated with higher levels of transduction in the murine retina than wild-type AAV2 and AAV2 (Y444F) injections (Fig. 1d–f).

### Increased humoral immune activation and T-cell infiltration into the retina following IVT of AAV2 (TM) vs. wild-type AAV2

**Fig. 2:**
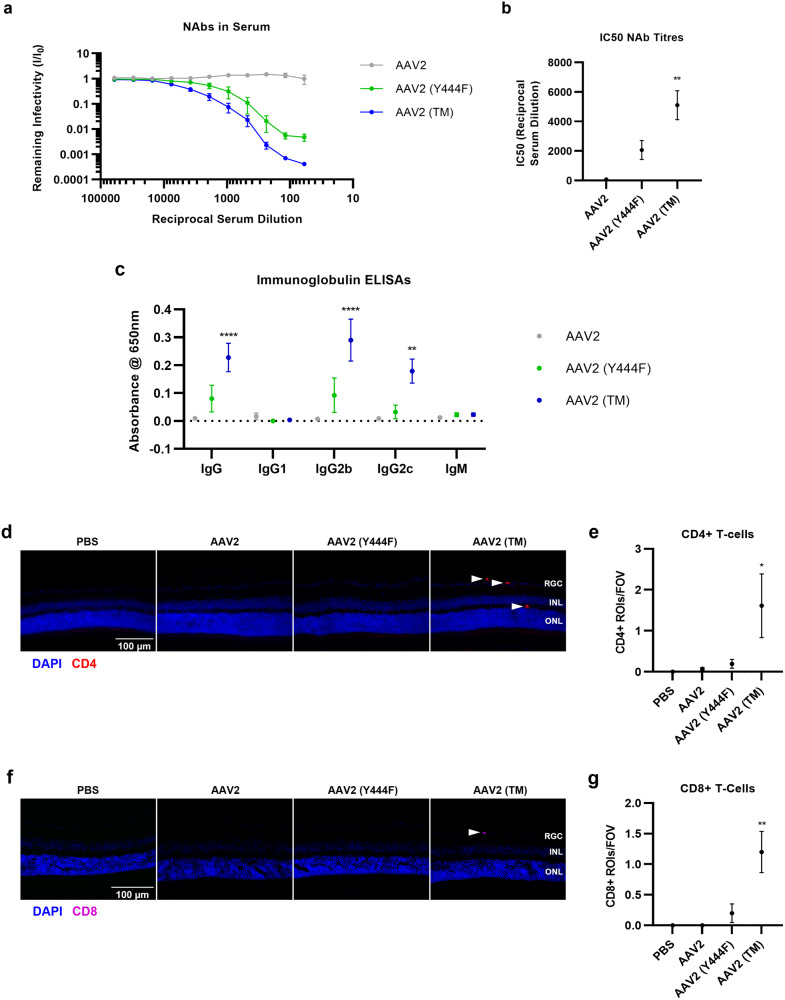
Intravitreal injection of a triple phosphodegron mutant AAV2 induces humoral and cellular adaptive immune responses. Vectors were injected and blood and tissue samples were taken three weeks later for analysis. All data is presented as a bar graph showing the mean value for each group ±SEM. All statistical analyses are vs. the AAV2 group. **a** Injection of phosphodegron mutant AAV2 increases murine sera neutralising antibody (NAb) levels. NAb titres were assessed using a HEK-293T/1E9 VP/mL AAV2.CMV.Luciferase system. Luminescence was measured across a range of dilutions of sera, and remaining infectivity was defined as the luminescent signal in each well divided by ‘Control’ values. **b** IC50 values for each group were calculated using non-linear regression (variable slope, four parameters) in GraphPad **p < 0.01, Brown-Forsythe ANOVA and Dunnett’s post hoc tests, *n* = 6. **c** Immunoglobulin ELISA assays were performed to assess the antibody subtypes responsible for the neutralising effect. **p < 0.01, ****p < 0.0001, two-way AVOVA and Dunnett’s posthoc tests, *n* = 6. **d** Representative 40x magnification epifluorescent microscope images showing that intravitreal injection of triple phosphodegron mutant AAV2 is associated with the presence of CD4+ T-cells in the retina. **e** Quantification of dataset was undertaken via manual counting of the number of CD4+ ROIs/FOV. *p < 0.05, **p < 0.01, Kruskal-Wallis and Dunn’s posthoc tests, *n* = 6–10. **f** Representative 40x magnification epifluorescence microscope images showing that intravitreal injection of triple phosphodegron mutant AAV2 is associated with the presence of CD8+ T-cells in the retina. **g** Quantification of dataset was undertaken via manual counting of CD8+ ROIs/FOV. *p < 0.05, **p < 0.01, Kruskal-Wallis and Dunn’s posthoc, *n* = 4–8.

First, the levels of NAbs were assessed in serum samples extracted 3wk post-IVT. Higher levels of neutralising activity were detected in mice who received AAV2 (Y444F) and AAV2 (TM) injections compared to wild-type AAV2 controls (Fig. 2a). These data were converted into IC50 values (dilution of serum that yields a 50% decrease in remaining infectivity), and this demonstrated increases in NAb levels following AAV2 (TM) compared with wild-type AAV2 IVTs (Fig. 2b). Next, TAb ELISA assays were performed to establish a correlation between the neutralising activity identified in the NAb assays and the presence of immunoglobulin isotypes known to participate in the anti-virus humoral immune response. Here, elevations in IgG, IgG2b and IgG2c titres were seen when comparing mice who received AAV2 (TM) and wild-type AAV2 IVTs, however, no changes in IgG1 and IgM isotype levels could be detected in this experiment (Fig. 2c). In summary, the results of the NAb and TAb assays suggested a higher level of humoral immune activation in AAV2 (TM) vs. wild-type AAV2 injected mice.

In the next set of experiments, eyes were extracted and processed into cryosections to assess whether the infiltration of CD4+ and CD8 + T-cells would be induced by IVT of wild-type and mutant AAV2. Increases in CD4 + T-cells were observed in the murine retina after AAV2 (TM) IVTs compared to mice who received wild-type AAV2 controls (Fig. 2d, e). Similar trends were evident in the CD8 + T-cell dataset, where elevated levels of infiltration were seen in AAV2 (TM) compared with wild-type AAV2 groups (Fig. 2f, g).

### Splenic lymphocyte populations are modulated by IVT of AAV2 (TM) vs. wild-type AAV2 vectors

**Fig. 3:**
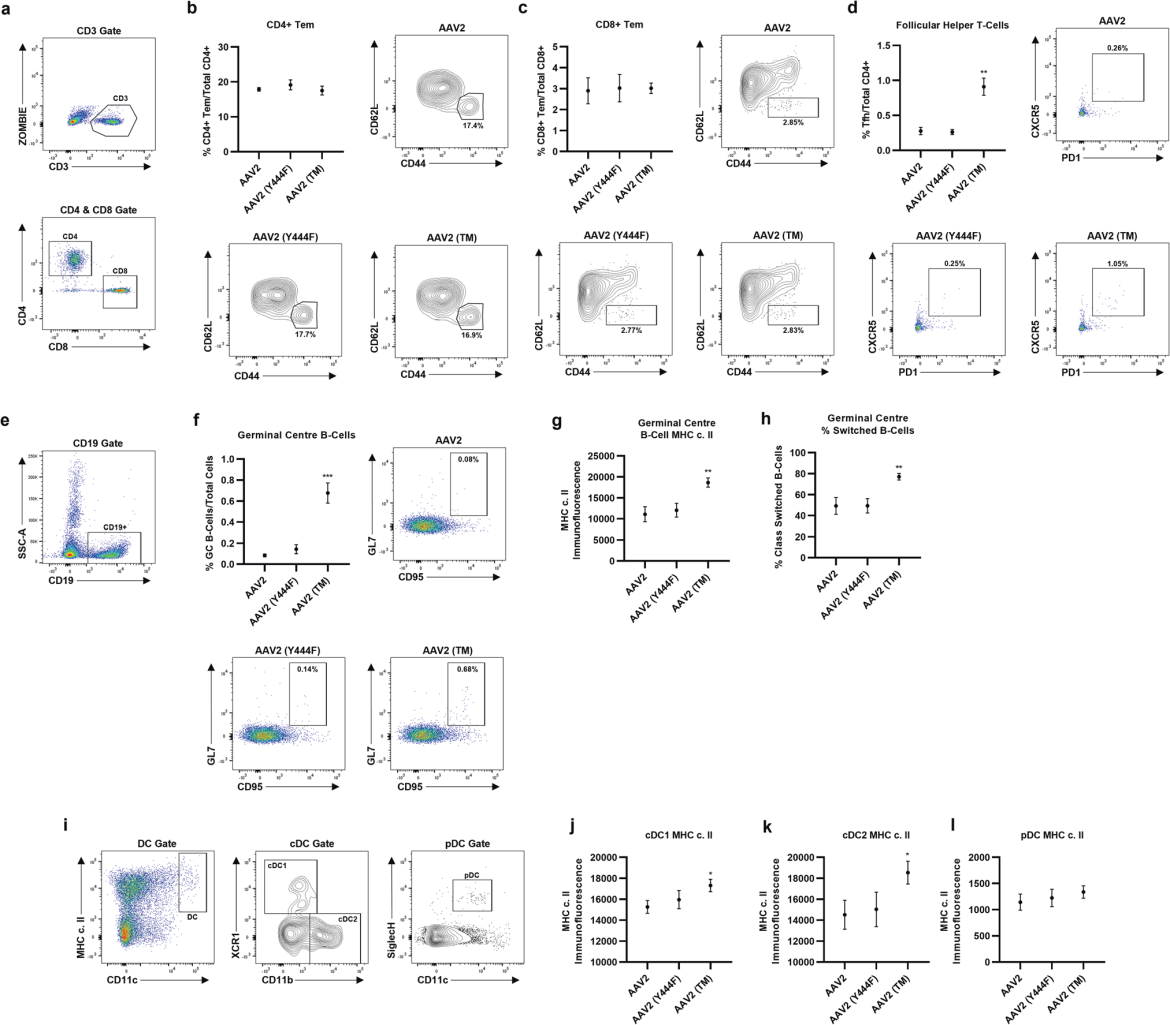
Intravitreal injection of AAV2 (TM) induces changes in splenic lymphocyte populations. Vectors were injected via IVT and spleens were harvested for analysis after three weeks. Changes were observed in the levels of follicular helper T-cells and germinal centre B-cells, and in classical and myeloid dendritic cell MHC c. II expression. All data is displayed as column graphs, and show the mean for each group ±SEM. All statistical analyses are vs. the AAV2 group. **a** CD4 and CD8 T-cell gating strategy used in subsequent analyses. **b** Effector memory CD4 + T-cell (CD3 + CD4 + CD8- CD62L_lo_ CD44_hi_) levels expressed as a percentage of total CD4 + T-cell levels, and representative flow plots. **c** Effector memory CD8 + T-cell (CD3 + CD4- CD8+ CD62L_lo_ CD44_hi_) levels expressed as a percentage of total CD8 + T-cell levels, and representative flow plots. **d** Follicular helper CD4 + T-cell (CD3 + CD4+ CXCR5_hi_ PD-1_hi_) levels expressed as a percentage of all CD4+ cells. **p < 0.01, Brown-Forsythe ANOVA and Dunnett’s T3 posthoc tests (*n* = 4), and representative flow plots. **e** B-cell gating strategy used for subsequent analysis. **f** Germinal centre B-cell (CD19+ IgM + /_lo_ IgD_lo_ CD95_hi_ GL7+ ) levels as a percentage of all splenic lymphocytes. ***p < 0.001, Kruskal-Wallis test and Dunn’s posthoc tests (*n* = 4), and representative flow plots. **g** Levels of MHC c. II expressed on germinal centre B-cells. **p < 0.01, one-way ANOVA and Dunnett’s posthoc tests (*n* = 4). **h** Percentage of class-switched B-cells (IgM_lo_ IgD_lo)_ in the germinal centres, as a percentage of all germinal centre B-cells. **p < 0.01, one-way ANOVA and Dunnett’s posthoc tests (*n* = 4), and representative flow plots. **i** Gating strategy used to delineate DC subsets for subsequent analysis **j** MHC c. II expression on conventional DCs subset 1 (cDC1) (CD11c^hi^ CD8a + XCR1 + ) dendritic cells. *p < 0.05, one-way ANOVA and Dunnett’s posthoc tests (*n* = 4). **k** MHC c. II expression on conventional DCs subset 2 (cDC1) (CD11c^hi^ CD11b+) dendritic cells. *p < 0.05, Student’s *t* test (*n* = 4). **l** MHC c. II expression on plasmacytoid (CD11c+ SiglecH+) dendritic cells.

Recent reports have suggested that IVT of AAV8 may be associated with the presence of vector genomes in the blood and spleen, suggesting that vector shedding from the ocular cavity may be responsible for humoral and cellular adaptive immune activation [13]. As such, we asked whether injection of phosphodegron mutant and/or wild-type AAV2 via IVT could be associated with changes in splenic lymphocyte populations pertinent to the generation of NAbs and retina infiltrating T-cells. First, spleens were extracted and cells gated by CD3 and CD4/CD8 for analysis of T-cell populations (Fig. 3a). No changes in the levels of effector memory CD4 (Fig. 3b) or CD8 (Fig. 3c) T-cell populations between AAV2 (TM), AAV2 (Y444F) and wild-type AAV2 groups were observed, however, an increase in follicular helper T-cell (Tfh) levels was evident when comparing AAV2 (TM) and wild-type AAV2 injected mice (Fig. 3d), a finding that may support the NAb and TAb data described above. Next, splenic samples were gated for CD19 to analyse B-cell populations (Fig. 3e). Germinal centre B-cell levels were assessed first as B-cells are known to proliferate and migrate to germinal centres when activated by antigen [14]. An increase in germinal centre B-cell levels was observed when comparing mice in the AAV2 (TM) group vs. the wild-type AAV2 group (Fig. 3f). Expression of major histocompatibility complex class II (MHC c. II) by germinal centre B-cells was then assessed to determine whether increased B-cell activation had been induced by administration of AAV2 (TM), and elevated MHC c. II immunofluorescence was evident in this group compared to wild-type AAV2 controls (Fig. 3g). Next, germinal centre B-cell class-switching was analysed, and an increase in ‘switched’ B-cells was seen in the AAV2 (TM) group compared to wild-type AAV2 injected mice, an observation that may corroborate the TAb data described above (Fig. 3h). Finally, conventional DC subsets, cDC1 & 2, and plasmacytoid DCs (pDCs) were investigated in terms of the level of MHC c. II immunofluorescence, a marker of DC activation [15]. First, DC subsets were gated according to MHC c. II, CD11c, CD11b, XCR1 and SiglecH expression (Fig. 3i). Analysis of MHC c. II immunofluorescence on cDC1 revealed elevated expression of this activation marker when comparing AAV2 (TM) and wild-type AAV2 mice (Fig. 3j). This trend was recapitulated in the cDC2 subset in which elevation of MHC c. II in the AAV2 (TM) vs. wild-type AAV2 groups could be seen (Fig. 3k). Finally, no changes in MHC c. II immunofluorescence was evident between any of the three groups analysed in the pDC subset (Fig. 3l). To summarise, analysis of splenic lymphocyte populations revealed changes in cells associated with the generation of systemic immunity to AAV2 capsids in the AAV2 (TM) groups compared with wild-type AAV2 injected mice, which may support the NAb/TAb and T-cell infiltration data outlined above in which immune activation was higher in the AAV2 (TM) group.

### IVT of AAV2 (TM) is associated with higher levels of retinal gliosis than wild-type AAV2 injections

**Fig. 4:**
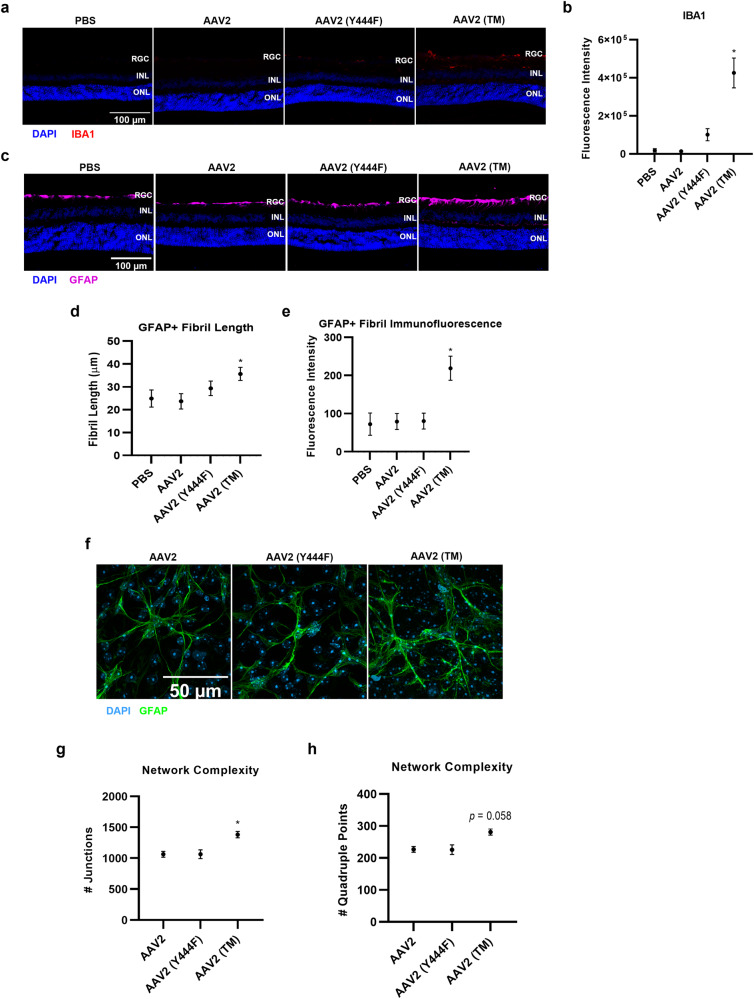
Intravitreal injection of a phosphodegron mutant AAV2 and high titre AAV2 leads to gliosis in the murine retina. Vectors were injected via IVT and retinas were extracted for analysis after three weeks. All data is presented as column graphs, with the mean value for each group shown ±SEM. All statistical analyses are vs. the AAV2 group. RGC, retinal ganglion cell layer; INL inner nuclear layer, ONL outer nuclear layer. **a** Representative 40x objective epifluorescent microscopy images of retinal cryosections showing IBA1 immunoreactivity in groups receiving phosphodegron mutant AAV2 injections. **b** Corresponding quantification performed in ImageJ shows the level of IBA1 immunoreactivity per field of view (FOV). *p < 0.05, **p < 0.01, Kruskal-Wallis ANOVA and Dunn’s posthoc test, *n* = 4–5. **c** Representative 40x objective epifluorescent microscopy images of retinal cryosections showing GFAP immunoreactivity in groups receiving phosphodegron mutant AAV2 injections. Corresponding quantification was performed using Simple Neurite Tracer, an ImageJ plugin (https://imagej.net/SNT). Each GFAP+ fibril was identified in each FOV and the length of each fibril (**d**) and its fluorescence intensity (**e**) and was measured. *p < 0.05, **p < 0.01, one-way ANOVA and Dunnett’s posthoc tests, *n* = 4. **f** Representative 63x images acquired on an Leiss Airyscan Confocal microscope showing increased complexity of astrocytic dendritic arbours in AAV2 (TM). Data was analysed in ImageJ using by assessing the number of junctions (**g**) and quadruple points (**h**) of skeletonized images. *p < 0.05, **p < 0.01, one-way ANOVA and posthoc Dunnett’s tests, *n* = 5–8.

Having identified a possible increase in systemic immune activation after IVT of AAV2 (TM) compared to wild-type AAV2 injections, we then tested whether retina glia cell populations may also be influenced by injection of the vectors. First, cryosectioned retina tissue was stained with Iba1 antibodies to analyse expression of a microglia activation marker [16]. In mice who were injected with AAV2 (TM), an increase in Iba1 immunoreactivity was observed vs. wild-type AAV2 injected mice (Fig. 4a, b). Next, GFAP antibodies were used to delineate Müller glia fibrils in cryosectioned retinae, which display increased length and intensity of immunoreactivity when exposed to proinflammatory stimuli [17]. AAV2 (TM) treated mice Müller glia fibrils displayed increased GFAP+ fibril length and immunoreactivity compared with mice in the wild-type AAV2 group (Fig. 4–e). Finally, GFAP antibodies were used to identify astrocyte dendritic arbours in flatmounted retina tissue, which may exhibit increased complexity in an inflammatory milieu [18]. An increase in the number of junctions and number of quadruple points in the dendritic arbours were observed in the AAV2 (TM) group compared to wild-type AAV2 control IVTs (Fig. 4f–h). In summary, this set of experiments showed that increased retinal gliosis may be associated with AAV2 (TM) vs. wild-type AAV2 IVTs, involving microglia, Müller glia and astrocyte cells.

### IVT of wild-type and phosphodegron mutant AAV2 vectors are not associated with detectable electrophysiological perturbations or loss of RGCs in the retina

**Fig. 5:**
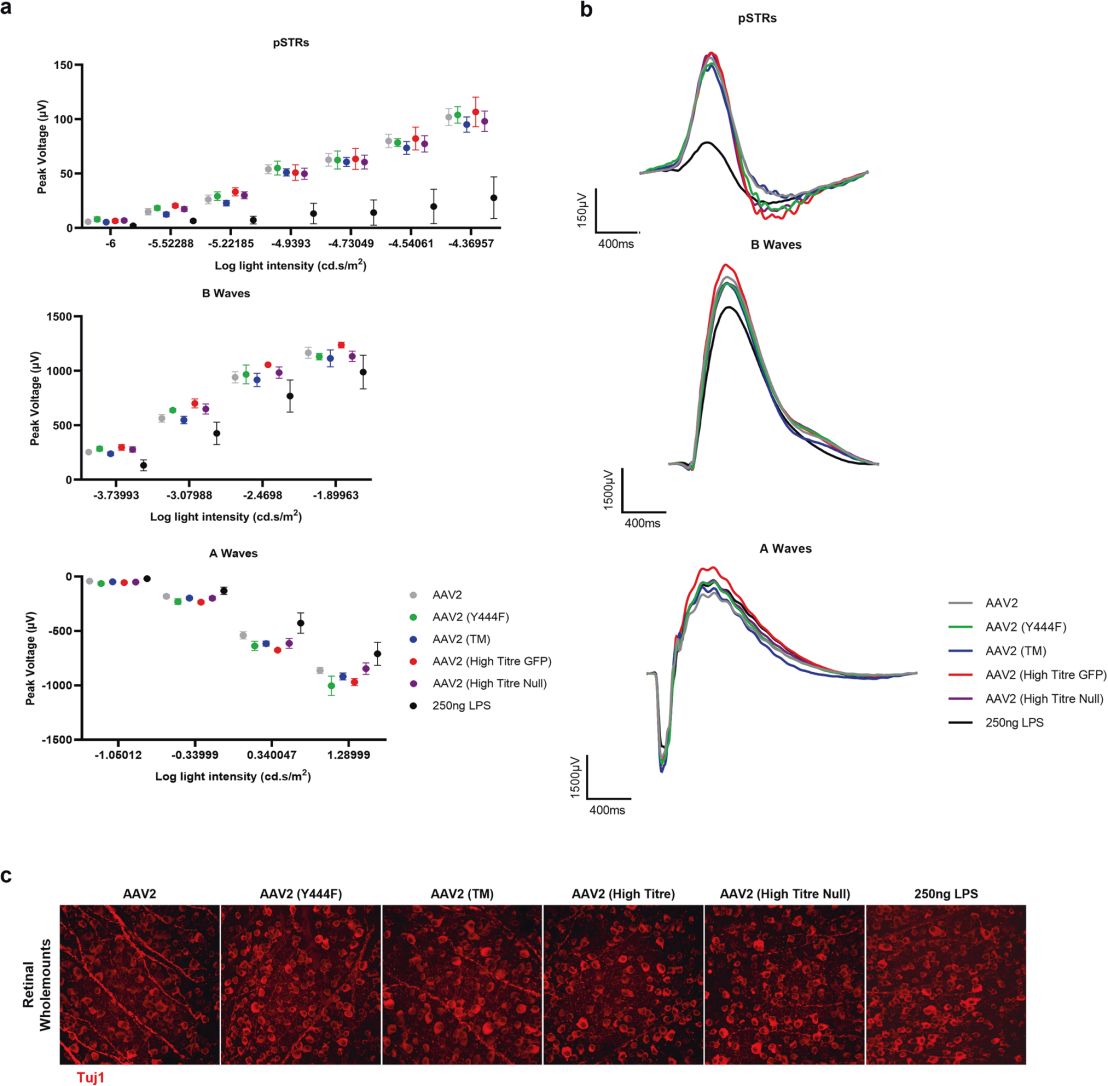
Injection of phosphodegron mutant vectors is not associated with changes in electrophysiological function in the murine retina. Vectors were injected via IVT and ERG was performed after three weeks. 250 ng of lipopolysaccharide (LPS) 24 h prior to ERG. **a** Average pSTRs, B-waves and A-waves from each treatment group (–4.37, –1.90 & 1.29 log cd.s/m^2^ light intensities, respectively). **b** Column charts showing peak voltages across a range of light intensities for each treatment group. Data is presented as the mean value ±SEM. **c** Representative 40x confocal images of Tuj1+ cells (an RGC marker) in flatmounted retina tissue.

The results outlined above demonstrate that AAV2 (TM) may elicit higher systemic and local immune responses than wild-type AAV2 vectors when injected into the vitreous cavity. To assess whether these immune responses could be associated with electrophysiological changes in the murine retina, ERG was performed and flatmounted retina tissue was stained with Tuj1 antibodies to identify possible loss of RGCs 3wk after vector administration. Assessment of ERG datasets showed no detectable changes between any of the vector IVTs across positive scotopic threshold (pSTR; RGC function), B-wave (rod bipolar cell function) and A-wave (rod photoreceptor cell function) recordings (Fig. 5a, b). Furthermore, there were no detectable changes in the number of Tuj1+ RGCs in the flatmounted retina tissue (Fig. 5c).

### Mutation of phosphodegron residues in AAV2 capsids is associated with reduced neutralisation by soluble HS and anti-AAV2 NAbs

**Fig. 6:**
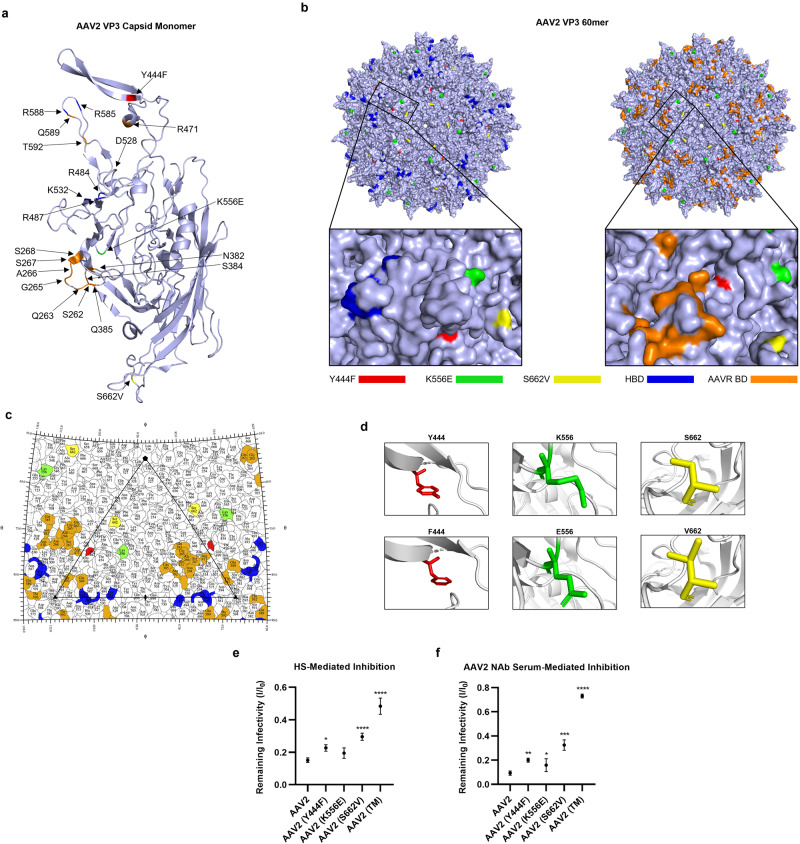
Mutation of AAV2 capsids at selected phosphodegron regions attenuates neutralisation by heparan sulphate (HS), and anti-AAV2 neutralising antibody-containing sera. All data is presented as bar graphs, with the mean value for each group shown ±SEM. All statistical analyses are vs. the AAV2 group. **a** VP3 capsid monomer showing the positioning of the three phosphodegron mutants, the five residues thought to mediate the binding affinity of AAV2 capsids to HSPG (blue) and the 14 residues thought to mediate binding of AAV2 to the AAV receptor (orange) (AAVR; KIAA0319). This model was generated in PyMol using 6ih9, a 2.8 Å resolution cryoelectron microscopy-derived structure of the AAV2 VP3 monomer. **b** AAV2 full 60mer capsid structure with highlighted phosphodegron mutations and heparin binding domains. Red = Y444F, Green = K556E, Yellow = S662V, Blue = R484, R487, K532, R585, and R588 heparin binding domains (HBDs), Orange = R471, D528, Q589, T592, S262, Q263, G265, A266, S267, N268, H271, N382 and Q385 AAVR binding domains (AAVR BDs). **c** RIVEM plot showing the positioning of the three phosphodegron mutants and their proximity to HBDs and AAVR BDs (using 6IH9 AAV2 coordinates, see (**b**) for colouring). **d** Schematic representation of mutations represented in PyMol. Mutations were introduced using PyMol’s Mutagenesis Wizard, and the optimal rotamer confirmation was selected in accordance with the software’s prediction. **e** Mutation of phosphodegron residues attenuates neutralisation by HS. AAV2 and mutant capsids were incubated with HS for 1 h, prior to addition to HEK293T cell media. Number of GFP+ cells was normalised to –HS controls for each vector group, allowing calculation of remaining infectivity (I/I0). *p < 0.05, ****p < 0.0001, one-way ANOVA and a Dunnett’s posthoc tests, *n* = 4. **f** Mutation of phosphodegron residues partially rescues neutralisation by AAV2 NAbs. AAV2 and mutant capsids were incubated with sera extracted from animals that had previously been injected intravitreally with AAV2, and the samples were tested to confirm the presence of NAbs. Calculation of remaining infectivity (I/I0) was performed as in (**d**). *p < 0.05, **p < 0.01, ***p < 0.001, **** *p* < 0.0001, one-way ANOVA and a Dunnett’s posthoc tests, *n* = 4.

Recent work has suggested that the incorporation of phosphodegron mutations into AAV2 capsids may elicit changes in binding affinity to the vector’s primary receptor, HSPG [1]. We hypothesised that phosphodegron mutant AAV2 vectors may also be more resistant to anti-AAV2 NAbs, possibly due to changes in NAb epitopes induced by conformational changes. Positioning of the three mutations was visualised in PyMol at the level of capsid monomers (Fig. 6a) and 60mer capsid oligomers (Fig. 6b). A RIVEM plot was used to determine whether the mutated residues lay proximal or distal to canonical HSPG binding residues, with AAV receptor binding amino acids included for reference (Fig. 6c). Rotameric conformation of the mutated residues was predicted in PyMol and this may have identified some slight variances with wild-type counterpart structures (Fig. 6d). First, we corroborated previous reports and demonstrated that mutants Y444F, S662V and TM exhibited attenuated neutralisation by soluble HS, which could suggest reduced binding affinity to HSPG (Fig. 6e). Next, we showed that remaining infectivity was increased in all single mutant capsid and AAV2 (TM) groups compared to wild-type AAV2, suggesting that the mutant vectors were more resistant to neutralisation by anti-AAV2 NAbs (Fig. 6f).

## Discussion

In this study, we examined the immune response arising from IVT of wild-type AAV2 and a triple phosphodegron mutant capsid, AAV2 (TM). Increased humoral immune activation and retinal T-cell infiltration were observed with the mutant capsid, in addition to changes in splenic germinal centre reactions and cDC1 & 2 activation. Elevated retinal gliosis was also increased in the AAV2 (TM) group, however, the immune responses identified were not associated with detectable electroretinography changes in the murine retina. Interestingly, AAV2 (TM) appeared to be less susceptible to neutralisation by soluble HS and anti-AAV2 NAbs.

To further understand how the mutation of AAV vectors affects the induction of the immune response, future investigations may seek to better understand how the attenuation of binding affinity to HSPG impacts vector shedding to the spleen and lymph nodes. Considering recent reports that HSPG binding affinity may affect extravasation of systemically administered vector into the retina [19], it is possible that AAV2 (TM) was better able to enter the circulation than wild-type AAV2. This model would be consistent with the elevation of cDC1 & 2 activation and increase in germinal centre reactions (B-cell levels, MHC c. II expression and class-switching) observed in this study, which naturally would lead to higher NAb and TAb titres. If increased vector shedding to the spleen and cDC1 activation with AAV2 (TM) did occur, then this may also explain the CD4+ and CD8 + T-cell infiltration dataset, as cDC1s are thought to participate in priming these immune cells [20].

An alternative explanation is that attenuated HSPG binding of AAV2 (TM) in the ILM may have affected interaction with microglia cells, perhaps due to increased permeation of vector particles into the retina, and increased microglia activation was observed when AAV2 (TM) and wild-type AAV2 were compared via Iba1 immunoreactivity in this study. Microglia have a demonstrated role in initiating immune responses in the CNS [21], which may include activation of retinal endothelium, a concomitant increase in vascular permeability, and increased extravasation of various immune cell types into the retina [22]. As such, our data may be explained by a model in which AAV2 (TM) IVT increased microglia activation and retinal vascular permeability, which increased shedding of vector protein and draining of capsid antigen to the spleen. Again, this model may be consistent with the cDC1 & 2 and germinal centre reactions observed, which increased NAb and TAb titres [23]. This explanation may also explain the patterns of T-cell infiltration into the retina, whereby AAV2 (TM) IVTs increased CD4+ and CD8+ counts by activating microglia cells which in turn elevated vessel permeability and leucocyte extravasation.

More research will be required to determine whether either of these mechanisms was responsible for the differences in immune activation observed between mutant and wild-type AAV2, or whether both pathways played a role. For instance, further work to characterise the interaction between microglia cells and blood vessel endothelium [22] after vector administration could prove a useful addition to the field. This could be complemented by investigations into whether microglia are capable of activating infiltrating CD4 + T-cells, which may drive development of a neuroprotective or neurodegenerative phenotype as reviewed in comparable CNS immunopathologies [24]. This information could prove useful in expanding upon the findings of the data presented, in which CD4+ and CD8 + T-cell infiltration was observed, yet the precise role the cells played at the site of infection was not studied. In particular, a more detailed characterisation of the CD8+ phenotype is warranted given the possibility that these cells killed AAV transduced hepatocytes in haemophilia B clinical trials, leading to diminished therapeutic benefit [25]. Obtaining this information may prove helpful in understanding why T-cell infiltration is commonly observed in ocular AAV gene therapy studies [12], but did not correlate with detectable changes in electroretinography in the present study.

In addition to microglia activation, we detected changes in GFAP+ Müller glia and astrocytes indicative of increased activation of these cell types in AAV2 (TM) vs. wild-type AAV2 injected retinae. Given the capacity of Müller cells to secrete proinflammatory cytokines across a range of retinopathies [26, 27] and in response to inflammatory stimuli [28], it is likely that the activated Müller glia identified in this study in some way participated in the anti-AAV2 innate immune response, possibly by facilitating development of an inflammatory microenvironment that assisted in leucocyte recruitment to the retina. The precise role that astrocyte activation played in the study is also unclear, however, future investigations into whether activation of infiltrating CD4 + T-cells was mediated by MHC c. II-restricted AAV2 antigen expressed by these cells, which has been described in other CNS neuropathologies [29], may be beneficial.

One interesting observation of the properties of AAV2 (TM) compared to wild-type AAV2 was that the vector may have been less susceptible to neutralisation by anti-AAV2 NAbs. As mentioned, pre-existing humoral immunity to capsid protein represents a key barrier to successful vector administration in ~50% of patients [30], which has led to the development of a number of strategies to circumvent the problem [31, 32]. Whilst we only utilised an in vitro neutralisation assay to demonstrate this effect, the data suggests that mutation of phosphodegron residues in AAV2 capsids may prove a useful, albeit partial, means of overcoming pre-existing NAbs in patients, highlighting a potential clinical utility for the technology in addition to improving retinal transduction [7] and increasing vector permeation to photoreceptor cells after IVT [1].

To summarise, after IVT of AAV2 (TM), increased humoral immune activation, T-cell infiltration, splenic germinal centre reactions and DC activation, and retinal gliosis was observed compared to wild-type AAV2 injections. However, it is important to note that the observed immune response was not correlative with changes in electrophysiology or loss of inner retinal cells. AAV2 (TM) transduced a greater proportion of retinal cells and was more resistant to anti-AAV2 NAbs, suggesting the capsid may be useful in overcoming pre-existing humoral immunity in patients. The present study therefore highlights novel aspects of phosphodegron mutant AAV2 immunobiology relevant to preclinical and clinical applications.

## Supplementary information

Supplementary materials

## Data Availability

All data needed to evaluate the conclusions in the paper are present in the paper and/or the Supplementary Materials. The published article includes all datasets and code generated or analysed during this study.
