# Supplementary material for: Immunobiology of a rationally-designed AAV2 capsid following intravitreal delivery in mice
Source: Gene Ther. 2023 Jun 29;30(9):723–35. doi: 10.1038/s41434-023-00409-x (PMC10506909; doi:10.1038/s41434-023-00409-x)
Supplement: Supplementary file 1 — Supplementary materials [file 41434_2023_409_MOESM1_ESM.docx]

Supplementary Materials for

**Immunobiology of a rationally-designed AAV2 capsid following intravitreal delivery in mice**

Michael Whitehead*, Andrew Sage, Andrew Osborne, Patrick Yu-Wai-Man and Keith R Martin

*Corresponding author. Email: [michael1](mailto:xxxxx@xxxx.xxx)993whitehead@gmail.com

**Fig. S1.**


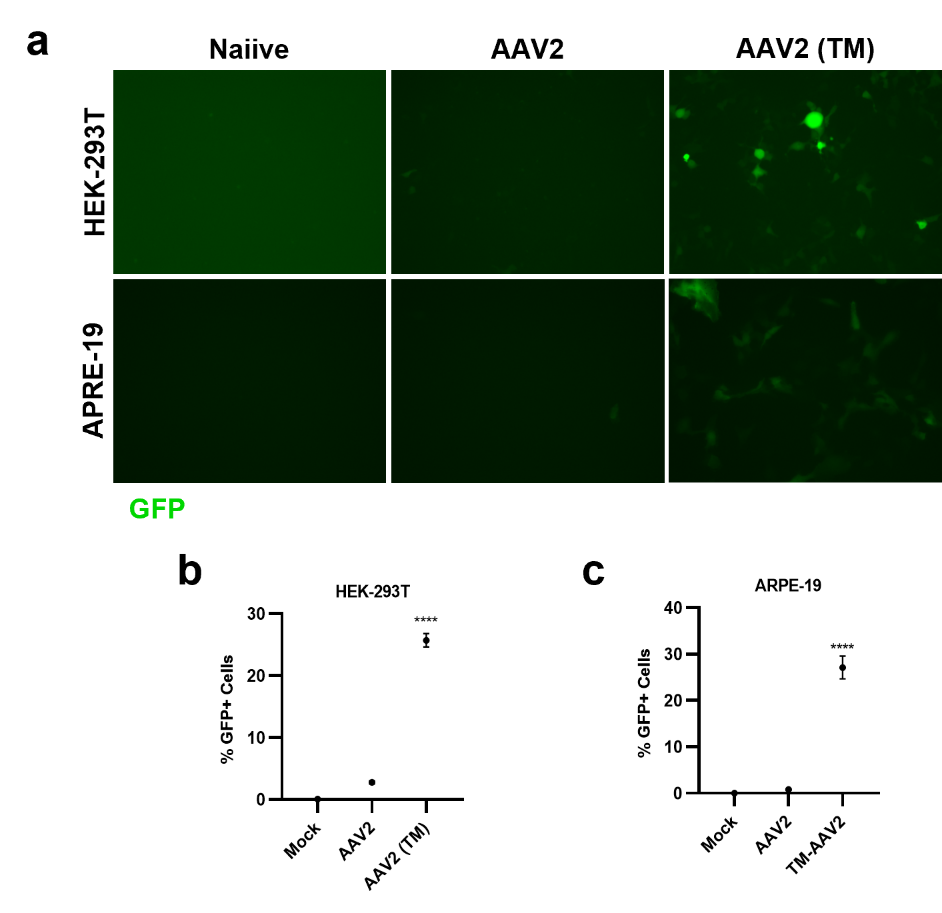


Fig. S1 - Transduction of AAV2 (TM) in vitro. (a) Representative images of GFP expression levels following a 24h incubation with the triple phosphodegron mutant AAV2 and WT AAV2 and Mock (DMEM-only). Here, 5E7 VP/mL was used for the HEK-293T cell line, 2E8 VP/mL for the ARPE-19 cell line. (b & c) Flow cytometry analysis of GFP expression in (b) HEK-293T cells and (c) ARPE-19 cells. A two-tailed Student’s t-test was used to assess statistical significance between groups. **** = *p* < 0.0001.

**Fig. S2**

Fig. S2.


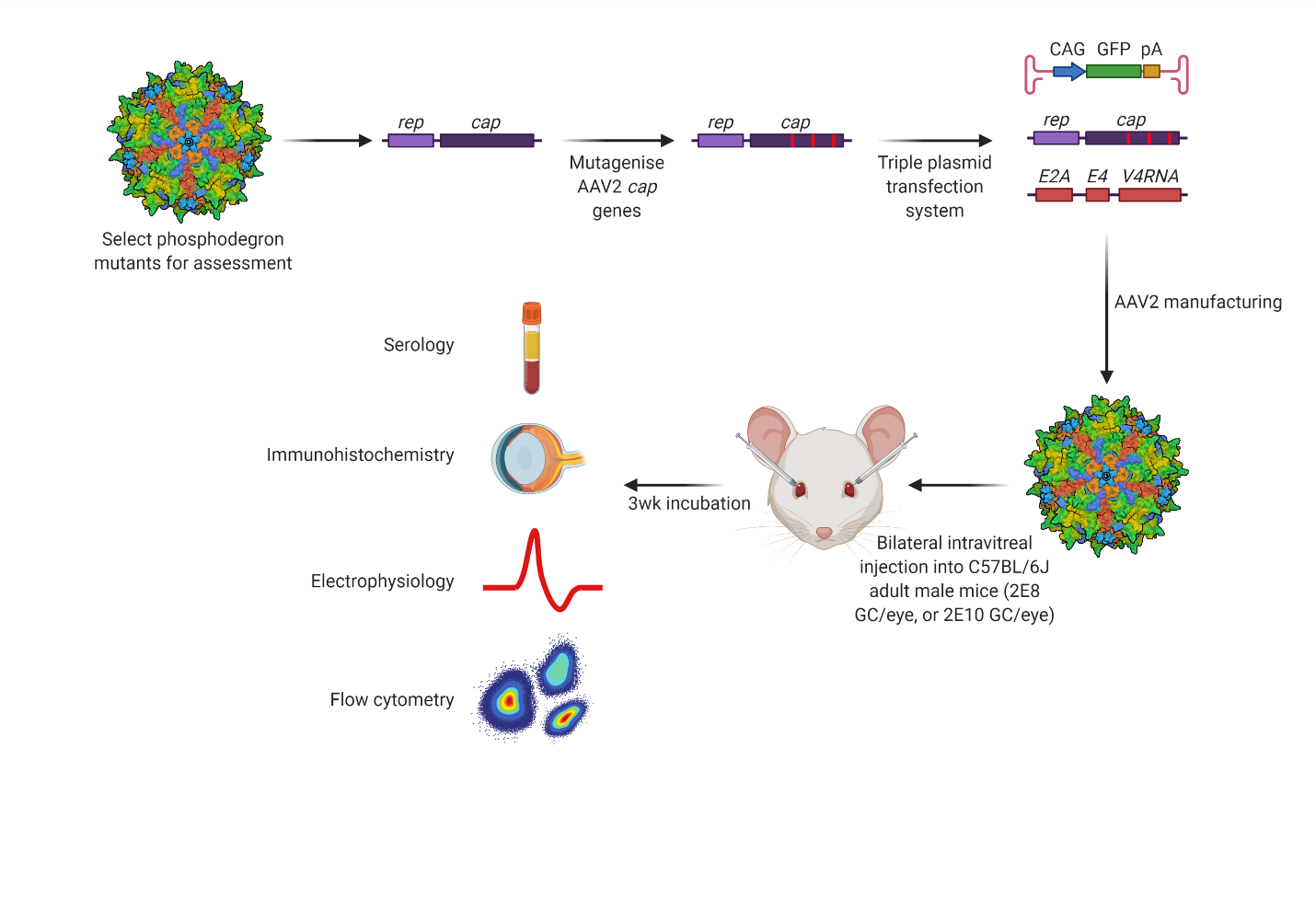


Fig. S2 – Phosphodegron mutations were selected for assessment and introduced into AAV2 capsids. Mice were injected bilaterally with 2E8 VG/eye. After three weeks, samples were collected for assessment after ERG.
